# Utilizing Rare-Earth-Elements Luminescence and Vibrational-Spectroscopies to Follow High Pressure Densification of Soda–Lime Glass

**DOI:** 10.3390/ma14081831

**Published:** 2021-04-07

**Authors:** Ferdinand Werr, Weniamin Yusim, Michael Bergler, Svyatoslav Shcheka, Armin Lenhart, Dominique de Ligny

**Affiliations:** 1 Institute of Glass and Ceramics, Friedrich-Alexander University Erlangen-Nürnberg, Martensstrasse 5, D-91058 Erlangen, Germany; Weniamin.Yusim@heinz-glas.com (W.Y.); michael.bergler@fau.de (M.B.); 2 Fakultät Werkstofftechnik, Nürnberg Institute of Technology Georg Simon Ohm, Wassertorstrasse 10, D-90489 Nürnberg, Germany; armin.lenhart@th-nuernberg.de; 3 Bayerisches Geoinstitut, University of Bayreuth, Universitätsstraße 30, D-95447 Bayreuth, Germany; slava.shcheka@mq.edu.au

**Keywords:** high pressure densification, residual stress, spectroscopic calibration, Raman spectroscopy, rare earth elements-luminescence, Brillouin spectroscopy

## Abstract

A new series of soda–lime glass naturally doped with Nd and doped with 0.2 wt% of Eu_2_O_3_ was densified in a multi-anvil press up to 21 GPa. The densities of the millimetric samples were precisely measured using a floatation method in a heavy liquid made with sodium polytungstate. The obtained densification curve is significantly different from the calibration previously reported, reaching a maximum densification saturation of 3.55 ± 0.14%. This difference could be due to better hydrostatic conditions realized in this new study. The densified samples were characterized using Raman and Brillouin spectroscopy, as well as the emission of both Eu^3+^ and Nd^3+^. The evolution of the spectra was evaluated using integration methods to reduce error bars. The relative precision of the calibration curves is discussed. The evolution of Nd^3+^ transition was found to be the most sensitive calibration. Linear dependence with the density was found for all observables, with exception for Brillouin spectroscopy showing a divergent behavior. The Brillouin shift shows an unreported minimum for a densification ~0.4%.

## Highlights:

1—Spectroscopic approach to detect the influence of permanent densification in glass.
2—Raman signal and REE are utilized as sensors.
3—Several calibration curves for these approaches are given.
4—Non-linear behavior of the Brillouin shift at early applied *P_max_*.

## 1. Introduction

In our modern life, glass is a frequently used material, e.g., as façade in buildings or displays of cars/handheld devices, due to its superior properties [1,2]. Anyhow, the great disadvantage of glass in comparison to other materials is its mechanical strength and its critical failure in the case of breakage due to its brittleness. To avoid overcritical loading in the final application and to prevent the glass product from breaking, knowledge of the local critical stress within the material is a crucial piece of information. Despite that, stress does not only show influence on the mechanical properties, but also affects the atomic structure and subsequently changes other properties significantly e.g., the index of refraction or density, among others. This impact on the material on a microscopic, but also on a global scale, makes it a highly interesting field of study from both an academic and engineering point of view. Experiments analyzing the indentation of glass showed that a pile-up volume around the indent is generated [3,4]. This plastic flow of material points out that even the common example of a brittle material shows plastic deformation. The stress field caused by the penetration is reported to generate local pressures of up to >10 GPa right underneath the tip of the indenter [3]. Local pressure values in this region cause a densification, dependent on the glass composition [5,6,7,8,9,10,11]. To investigate a micrometric formation like an indent, or a specific region of interest, spectroscopic methods are especially suitable [12]. The final assessment of a property, e.g., density, is highly dependent on the calibration used, as it is establishing the link to the spectroscopic observable. Despite its very large interest and overall present usage, only few studies report a relation between the maximal pressure reached and the densification for soda–lime–silicate glass [6,10,11,13,14]. In the past, Cr-luminescence was utilized as pressure sensor to study indents and Nd-luminescence to study crystals [15,16,17,18]. Rare-earth element (REE)-luminescence was disregarded for glass, even though their electronic structure makes them an ideal intrinsic sensor. The purpose of this article is to provide several calibration curves to assess the local pressure history and densification of glass products at a region of interest by utilizing spectroscopic methods, namely Raman, Brillouin and Nd^3+^- as well as Eu^3+^-luminescence. We will compare these different approaches by means of their sensitivity at the various pressure regions, i.e., their applicability, their overall pressure-dependent behavior and characteristic advantages and disadvantages. Detailed structural interpretation of the observed spectral variations is outside of the scope of this article. An in-depth discussion regarding the structural origin of the variations is going to be made in a separate report. The proposed characterization methods allow for the use of spectroscopic equipment, which is uncomplicated to operate and whose spectral acquisition is comparably easy to handle. Insight on material properties can be obtained at a local region of interest with a high spatial resolution of few micrometers, e.g., mappings of indentations, inclusions, or laser modifications. The use of rare-earth elements luminescence reduces the acquisition time of large-scale mappings drastically, since a full high-quality spectrum can be obtained within seconds or even milliseconds.

## 2. Sample Preparation

### 2.1. Eu_2_O_3_ Doping of Commercial Low-Iron Soda–Lime Float Glass

Commercially available soda–lime–silicate glass OptiWhite^TM^ (Pilkington Ltd., Lancashire, UK/NSG Co., Ltd., Tokyo, Japan; Composition [mol %]: 71.33 SiO_2_, 12.37 Na_2_O, 9.25 CaO, 6.29 MgO, 0.35 Al_2_O_3_, 0.25 K_2_O, 0.15 SO_3_, 0.01 Fe_2_O_3_) was used as sample material, which already contains Nd_2_O_3_ in traces introduced by the raw materials. The glass was ground in an agate mortar, mixed thoroughly with addition of 0.2 wt% of Eu_2_O_3_ and remolten at 1570 °C for 1.5 h. After casting on a brass plate, the glass was annealed at 559 °C for 1 h and left for cooling to room temperature overnight to assure minimum remaining residual stress. Eventually, a visually transparent and colorless glass was obtained. Six cylinders of 3.8 mm diameter were drilled from the glass block, cut to a length of 5 mm and their front faces were polished to reduce the risk of cracking in the later densification process. An additional, smaller cylinder was prepared from the same block of pristine glass to repeat the same 19 GPa densification as the original sample got in contact with the MgO octahedron of the densification setup (more detailed information in the following section).

### 2.2. Inducing Permanent Densification at Room Temperature

**Figure 1:**
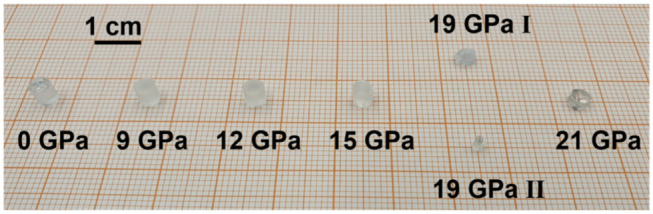
Overview of the series of permanently densified glass cylinders; densification was done at room temperature using a multi-anvil press (MAP) at maximum pressures of 9 GPa, 12 GPa, 15 GPa, 19 GPa and 21 GPa; preparation of the 19 GPa sample was repeated with a smaller glass cylinder (19 GPa II) as the original cylinder (19 GPa I) came into contact with MgO in the experimental setup.

Densification of the glass cylinders was done at room temperature in a 50 MN/5000 ton (ZwickRoell GmbH & Co.KG, Ulm, Germany) multi-anvil press (MAP) to permanently densify the glass hydrostatically without relaxation. The setup consisted of a partially-sintered MgO octahedron, in which a millimetric sample compartment was drilled. In this compartment, the glass sample was completely embedded in low-friction NaCl to avoid any possible contact with the MgO. Even though MgO is already quite considerable, low-friction NaCl is a superior pressure transducing medium and therefore was chosen to prevent shear stress. This stress recipient convolute was centered in the cavity of the eight cornered truncated tungsten carbide cubes. In the next step, the complete setup was put in a cubic cavity formed by six steel anvils, which are arranged in hemispheres in the guide blocks of the press, and compressed, eventually. For further and more detailed information on the equipment, consider various literature references [19,20]. Loading was done continuously in four hours to the target maximum pressure with a dwell time of two hours to ensure that the material was given enough time to respond to the applied *P_max_*. Decompression cycle was set to eight hours to reduce the risk of breaking. Seven millimetric sized samples were prepared in this procedure with a target maximum pressure *P_max_* of 0 GPa, 9 GPa, 12 GPa, 15 GPa, 19 GPa I, 19 GPa II and 21 GPa (Figure 1). The error bars of the applied pressure were estimated to be ±1 GPa. Due to the uniform hydrostatic compression conditions, the samples were affected by much less shear stress in comparison to diamond anvil cell (DAC) experiments. The samples with 0 GPa to 15 GPa were without visible cracks, while the sample of 21 GPa broke in half. The high-pressure preparation of 19 GPa was redone with a smaller cylinder (19 GPa II) as the original cylinder got in contact with the MgO octahedron while densification. The embedded MgO particles were removed carefully by manual polishing and the sample was used to estimate the reproducibility of the densification process. As the 19 GPa I sample may have been non-conforming, the data value was excluded in any calibration related to *P_max_* as it could have altered the fitting, but was included in calibrations related to the density as it is a constitutive material property.

## 3. Experimental Methods

### 3.1. High Precision Density Determination

If a solid is put into a liquid it will either sink (*ρ_liq_ < ρ_solid_*), swim (*ρ_liq_ > ρ_solid_*) or float (*ρ_liq_ = ρ_solid_*) in the liquid. The sink–float technique utilizes this very basic behavior and is especially well suited for high precision density determination of small samples [7].

(1) $$\rho_{heavy\;liquid\;=\frac{m_{Air}-m_{heavy\;liquid}}{m_{Air}-m_{H_{2}O}}\;*\;\rho_{H_{2}O}}$$

With Equation (1), where *m_Air_*, *m_H_*_2*O*_ and *m_heavy liquid_* are the mass of a reference weight in the corresponding media and *ρ_H_*_2*O*_ is the density of water at corresponding temperature, the density of the liquid and subsequently, in the case of flotation, the density of the sample can be determined. Sodium polytungstate (SPT) was dissolved in distilled water to obtain a heavy liquid with an initial density of ~2.9 g/cm^3^. The density of the heavy liquid can be easily adjusted by either adding distilled water to lower the density or adding stock initial heavy liquid to increase its density. This procedure was repeated until flotation of the sample was accomplished and observed for 20 min to assure an equilibrium of the density liquid. In addition, we monitored possible temperature changes with an accuracy of 0.5 °C and a stainless steel cube, functioning as the reference weight, was kept inside the liquid at all time to minimize the influence of a temperature gradient. Two samples, e.g., the 21 GPa and the 19 GPa I cylinder, were observed in the liquid at the same time to ensure certainty of separation inside the liquid and to consequently detect density variations between each other. Compared to the conventional Archimedes method, the sink–float technique does not longer rely on the mass of the sample, but on the mass of the reference weight. This guarantees a very high precision density determination, as the uncertainty of the balance (*m_Reference_ >> m_Sample_)* affects the measurement less. We estimated the accuracy of the sink–float technique was three times higher in comparison to the conventional Archimedes method, resulting in an uncertainty of ±0.002 g/cm^3^.

### 3.2. Acquisition and Data Treatment of the Raman-, Brillouin- and Luminescence-Spectra

#### 3.2.1. General

To give access of application to the reader, the spectroscopic data evaluation is going to be highly detailed. All spectroscopic experiments were done in backscatter geometry, which means the observed signal of the material was parallel to the excitation incidence. Presented data points are the statistical mean value of several measurements at different positions to better reflect possible inhomogeneities and provide a statistical evaluation. The given error bars were chosen in a size which covers both the equipment-related uncertainty, as well as the standard deviation of the combined measurements. The eventually determined data values are listed in Table A3 (*centroid σ*) and Table A4 (*mean* $\bar{I}$) in the Appendix A.

#### 3.2.2. Raman and Brillouin Experimental Setup

For characterization of the glass structure an equipment that was developed in-house was used. The device called ARABICA (***A**ssociated**Ra**man**B**rillouin**Ca**lorimeter*) combines the techniques of Raman-spectroscopy, Brillouin-spectroscopy, and calorimetry in one setup. With this equipment, it is possible to record atomic vibrational information in-situ while performing calorimetric studies on the material. A detailed explanation of its capability is described elsewhere [21]. A 488 nm Ti-Saphire CW-laser is used as excitation source in combination with a ×50 objective/numerical aperture 0.42, which results in an estimated focal spot size diameter of 1.4 µm and depth of field 11.1 µm. An iHR 320 Horiba monochromator combined with a Sincerity UV-VIS CCD camera was utilized to record the Raman signal. The setup allows an acquisition with a resolution of ±0.5 cm^−1^ and gives the possibility of detecting small spectral variations with high precision. A single measurement is the combination of seven exposures collected with 180 s exposure time. Brillouin spectra were recorded simultaneously at the same position as the Raman spectra with a tandem Fabry–Perot interferometer TFP-2 HC from JRS Scientific Instruments with an accuracy of ±0.03 GHz.

#### 3.2.3. Raman Data Treatment

**Figure 2:**
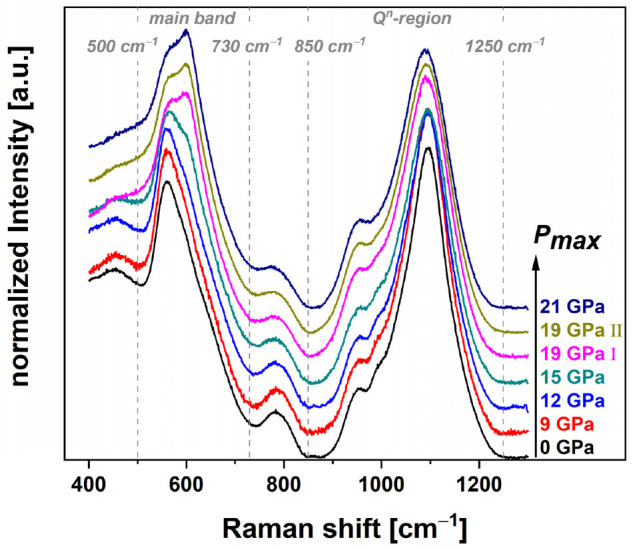
Raman spectra (400 cm^−1^–1300 cm^−1^) of the densified cylinders with increasing maximum pressure; visible variations of the main band from 500 cm^−1^–730 cm^−1^ are related to the evolution of the Si–O–Si inter-tetrahedra angle distribution [9,22]; the Q^n^-region (850 cm^−1^–1250 cm^−1^) is assigned to Si–O stretching vibrations [25].

**Figure 3:**
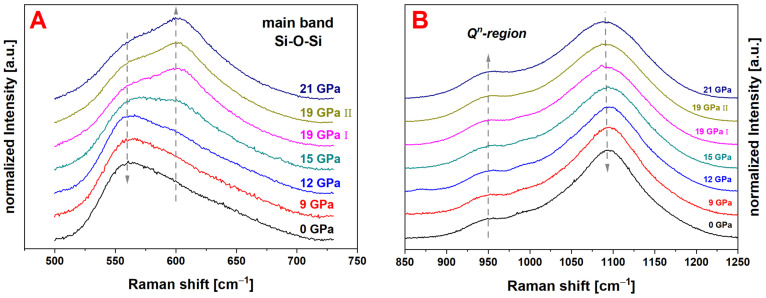
Raman spectra of the main band (**A**) related to Si–O–Si inter-tetrahedra angles of the permanently densified glass series (0 GPa–21 GPa); the contribution at ~560 cm^−1^ decreases while the contribution at ~600 cm^−1^ increases over the experimental series; spectra of the high frequency region related to the Q^n^-species (**B**); a ratio change of Q^3^- to Q^2^-species is observed; the large band attributed to Q^3^-species at ~1080 cm^−1^ shows a decrease and a shift of the peak position to lower wavenumbers; the Q^2^-species related band at ~950 cm^−1^ increases as well as the less pronounced Q^4^-band at ~1200 cm^−1^; to evaluate the spectral variations the main band as well as the Q^n^-region was evaluated using the centroid parameter according to Equation (2).

Raman spectra were acquired in the range between 6 cm^−1^ to 1530 cm^−1^. To assure a correct frequency position of the Raman spectra, CaCO_3_, with its known peak positions at 154.9 cm^−1^, 281.2 cm^−1^, 712.4 cm^−1^, 1086.2 cm^−1^ and 1435.8 cm^−1^ was used as a reference material, to rescale the spectra to the proper frequency position. The peak positions were determined as the arithmetic mean of several fitted calcite Raman spectra from the publicly available RRUFF database. In this way, a drift or misalignment of the frequency position of the detector can be eliminated. The rescaled spectra were cut below 400 cm^−1^ and above 1300 cm^−1^ to reduce influence on the linear background subtraction, with the anchor points set at 850 cm^−1^ and 1250 cm^−1^, and the normalization in respect to the total area (Figure 2). The so-called main band, related to vibrations of the inter-tetrahedra angles, was extracted in the range of 500 cm^−1^ to 730 cm^−1^ to further evaluate this region separately. Again, a linear background was applied at the endpoints and the spectra were renormalized to the total main band area (Figure 3A). Eventually, the centroid parameter *σ* (Equation (2)) of the main band was calculated (for further explanation see corresponding section). The methodology of data treatment is in accordance as proposed by Deschamps et al., who investigated soda–lime glass in high pressure DAC experiments [9]. A linear behavior of the bands located at 560 cm^−1^ and 600 cm^−1^ with increasing pressure is reported, which makes the main band a suitable feature for pressure calibration [9,22]. The described evaluation method is used in other published articles discussing similar topics [7,23]. Furthermore, the high-frequency region related to the Q^n^-species was evaluated separately by extracting the region between the anchor points at 850 cm^−1^ to 1250 cm^−1^ and a renormalization to the Q^n^-region area (see Figure 3B). Spectral variations were observed in the same way using the centroid, as aforementioned. The global decrease of the centroid contains several phenomena happening at the same time. The position of the main Q^3^ contribution at ~1080 cm^−1^ shifts to lower frequency. This shift is opposed to the shift of the main band and in agreement with the central force model of Sen and Thorpe [24]. A simultaneous evolution of the Q^n^-units population is also taking place. The contribution of the Q^3^-species, is translated to Q^2^-species, related to the vibration at ~950 cm^−1^, as well as a decrease of Q^4^-species at ~1200 cm^−1^ [25]. This behavior suggests that the regular disproportion reaction, 2Q^3^ = Q^2^ + Q^4^, is not followed. Furthermore, a global broadening of the spectra is evident, which may signify an increase of disorder.

#### 3.2.4. Brillouin Data Treatment

**Figure 4:**
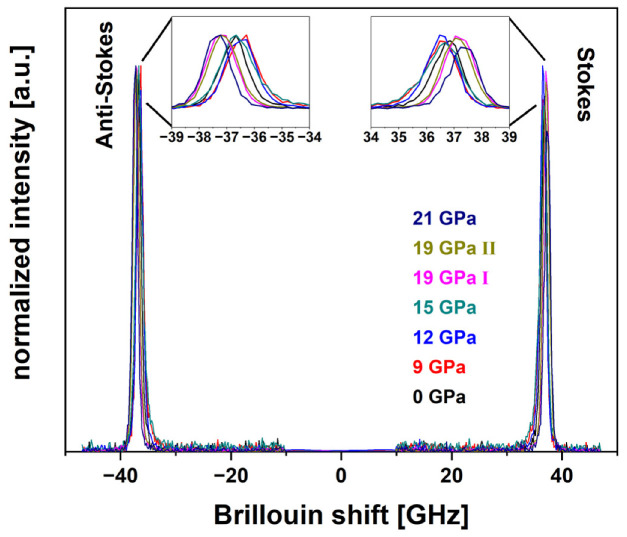
Brillouin spectra of the permanently densified cylinder series with *P_max_* of 0 GPa, 9 GPa, 12 GPa, 15 GPa, 19 GPa I, 19 GPa II and 21 GPa; the insets highlight the evolution of the peak positions.

For better display and comparability, in Figure 4 the Brillouin spectra of all the samples were set to zero and normalized. The dominant Rayleigh peak located at 0 GHz in the Brillouin spectra was removed to evaluate the Stokes and anti-Stokes contributions. To extract the value of the longitudinal Brillouin shift, a Gaussian was fitted to both contributions and their statistical mean position was calculated. The insets in Figure 4 show both Stokes and anti-Stokes observable changes. The occurrence of these peaks is linked to the interference of the sampling laser excitation with the acoustical vibrations of the atoms. Depending on the location of the Stokes and anti-Stokes contribution, insight on the acoustical properties can be drawn. Brillouin spectroscopy is therefore sensitive to structural variations of the glass and correlates to the long-range elastic properties, e.g., sound velocity, of the material [8,26].

#### 3.2.5. Nd^3+^- and Eu^3+^-Luminescence Acquisition

A commercial Thermo Scientific Nicolet^TM^ Almega^TM^ µ-Raman, containing two excitation lasers with a wavelength of 532 nm (pumping of Eu^3+^-luminescence) and 780 nm (pumping of Nd^3^^+^-luminescence) were used for acquisition of the REE-luminescence spectra. An objective with a ×100 magnification and a numerical aperture of 0.9 was used with an estimated focal spot size diameter of 0.7 µm and a depth of field of 2.6 µm for the 532 nm laser and a diameter of 1.1 µm and depth of field of 3.9 µm for the 780 nm, respectively. An accuracy of ±1.0 cm^−1^ for the frequency position was determined. A single measurement consisted of ten integrated exposures each with an equivalent exposure time of 100 milliseconds for the Eu^3+^-luminescence as it was necessary to use only a tenth of the excitation laser power to avoid detector saturation, due to very efficient pumping conditions. For the Nd^3+^-luminescence, the acquisition parameters were adjusted to five exposures each with an increased exposure time of 120 s to maintain recording of a high-quality spectrum.

#### 3.2.6. Neodymium Data Treatment

**Figure 5:**
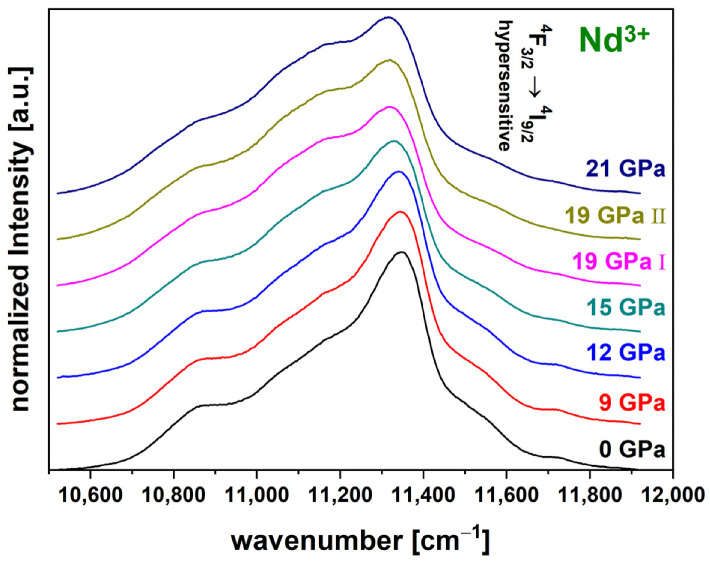
Luminescence spectra of the hypersensitive transition ^4^F_3/2_ → ^4^I_9/2_ of the Nd^3+^-luminescence for the permanently densified glasses (0 GPa to 21 GPa); spectra were acquired with a 780 nm laser; a clear broadening of the left shoulder next to the maximum peak can be seen and consequent shifting of the centroid position to a lower wavenumber.

The luminescence spectra of the Nd^3+^ were collected in the range between 9320.5 cm^−1^ to 12,678.5 cm^−1^. The region between 10,520 cm^−1^ and 11,920 cm^−1^ was extracted from the raw spectra and further evaluated (Figure 5). An accurate frequency position was maintained by monitoring the frequency of the excitation laser and routinely recalibrating the spectrometer. The spectrum in this region corresponds to the hypersensitive transition ^4^F_3/2_ → ^4^I_9/2_, which makes it especially suitable since it reacts to atomic environmental influences, e.g., stress and densification [15,27]. A linear background was removed with the anchor points being fixed at the endpoints of the spectra and a normalization to the total area was applied before plotting. To evaluate the variations of the Nd^3+^-luminescence, the previously mentioned centroid was obtained in the same manner (Equation (2)). With increasing pressure, the shoulder to the left of the main peak broadens and subsequently shifts the centroid position to lower wavenumbers.

#### 3.2.7. Europium Data Treatment

**Figure 6:**
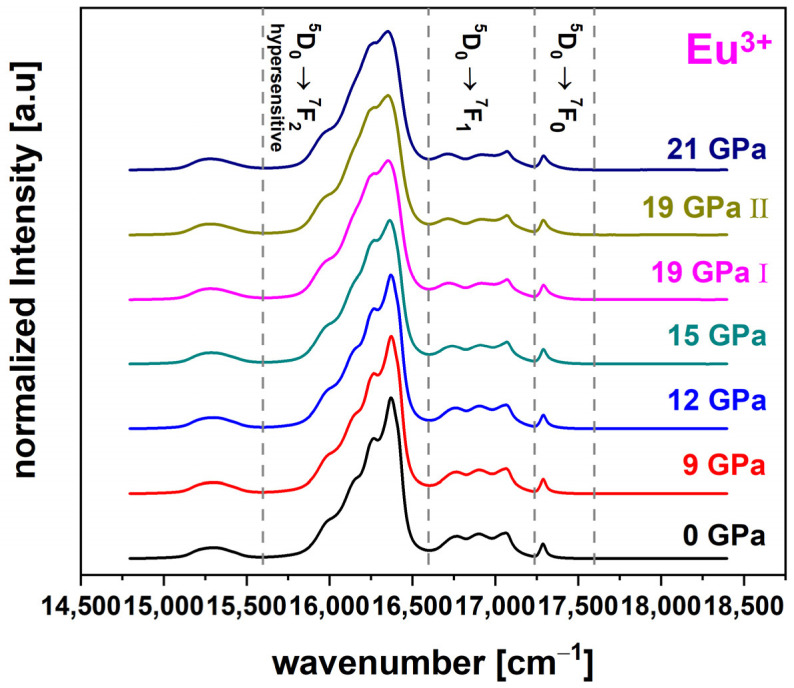
Exemplary luminescence spectra of the ^5^D_0_ → ^7^F_0_ (17,237 cm^−1^–17,597 cm^−1^), ^5^D_0_ → ^7^F_1_ (16,597 cm^−1^–17,237 cm^−1^) and hypersensitive ^5^D_0_ → ^7^F_2_ (15,597 cm^−1^–16,597 cm^−1^) transition of the Eu^3+^-luminescence for the permanently densified glass (0 GPa to 21 GPa); spectra were acquired with a 532 nm laser and the shown frequency bands were used as anchor points for evaluation.

Luminescence spectra of Eu^3+^ were acquired in the region of 15,297 cm^−1^ to 18,655 cm^−1^ (Figure 6). Furthermore, data processing of the Eu^3+^ is equivalent to the one previously described for Nd^3+^, however the frequency range had to be adjusted accordingly. The spectrum contains three emissions which corresponded to the transitions ^5^D_0_ → ^7^F_0_ (17,237 cm^−1^ to 17,597 cm^−1^), ^5^D_0_ → ^7^F_1_ (16,597 cm^−1^ to 17,237 cm^−1^) and ^5^D_0_ → ^7^F_2_ (15,597 cm^−1^ to 16,597 cm^−1^) [28]. The ^5^D_0_ → ^7^F_2_ transition shows the characteristics of a hypersensitive transition as well, which makes comparison to the Nd^3+^-luminescence particularly appropriate. As the ^5^D_0_ → ^7^F_0_ transition shows no splitting of the energy levels, it can be assumed that a spectral variation is a direct result of the evolution of Eu^3+^-ion position distribution [28]. Again, Equation (2) was utilized to evaluate the spectral variations.

### 3.3. Characterization of Spectral Variations

Equation (2) is the mathematical function which was used to evaluate the spectra according to the *centroid parameter* *σ*, proposed by Deschamps et al. [9]. *σ* describes as the equal-area position at which the area between the determined limits underneath the graph is exactly half of the complete integrated area. This is a reliable parameter for evaluating spectral variations as it is sensitive to intensity ratio changes, broadening and asymmetry of the spectrum. The limits used for evaluation of each spectroscopic analysis are given in the experimental section.

(2) $$\frac{1}{2}=\;\frac{\int_{\omega 1}^{\sigma }I_{spectra}\left(\omega \right)\;d\omega }{\int_{\omega 1}^{\omega 2}I_{spectra}\left(\omega \right)\;d\omega }$$

Even if this unique coefficient will be used in this paper, other evaluation parameters such as the *mean* $\bar{I}$ (Equation (3)), which is defined by Hehlen, and the different distribution momentum can be qualitatively equivalent evaluation methods [29]. For instance, the calibrations in terms of the *mean* $\bar{I}$ are given in the Appendix A, also (Table A1 and Table A2) and was shown to be linearly correlated to *σ*.

(3) $$\bar{I}=\;\frac{\int_{\omega 1}^{\omega 2}\omega \cdot I_{spectra}\left(\omega \right)\;d\omega }{\int_{\omega 1}^{\omega 2}I_{spectra}\left(\omega \right)\;d\omega }$$

## 4. Results & Discussion

### 4.1. Densification of Glass Cylinders

**Figure 7:**
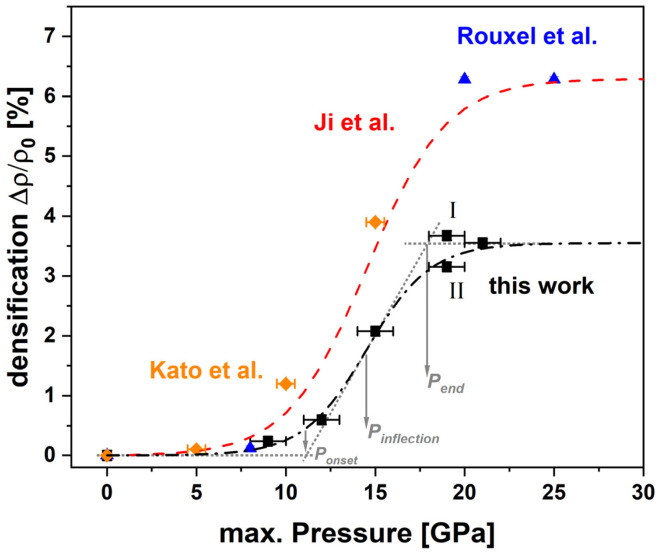
Percentage of densification [%] (***ρ_pristine_***_***glass***_ = 2.505 g/cm^3^/±0.002 g/cm^3^) over increasing maximum pressure (0, 9, 12, 15, 19 I, 19 II, 21 GPa/±1.0 GPa) of hydrostatic compressed soda–lime–silicate glass; gray dashed lines and arrows indicate the location of *P_onset_*, *P_inflection_* and *P_end_*; black data ≙ this study and its corresponding sigmoidal fitting (Equation (5)); the linear approach is giving a *P_onset_* = 10.9 ± 0.8 GPa, *P_end_* = 18.1 ± 0.8 GPa and *P_inflection_* = 14.5 ± 0.3 GPa with *m* = 0.14 ± 0.02; the maximal variation Δ was determined at 3.55 ± 0.14%; studies by Rouxel et al. (blue) and Ji et al. (red dashed line) report an earlier *P_onset_* = 10.1 GPa and a significant higher saturation at 6.3% after *P_end_* = 19 GPa; *P_inflection_* is determined at the same value of 14.5 GPa with a slightly lower slope *m* = 0.11; studies by Kato et al. (orange).

For better comparison with the literature (red dashed line, Ji et al.; blue datapoints, Rouxel et al.; orange datapoints, Kato et al.), the measured densification (black datapoints) over increasing maximum pressures is shown in Figure 7 [6,7,10]. It was calculated as the increase of density over the initial density Δ*ρ*/*ρ*_0_. The initial density of the pristine glass was determined at 2.505 g/cm^3^. The first sample densified with 9 GPa maximum pressure already showed an increase in density to 2.511 g/cm^3^ which is equivalent to 0.24% densification. The densification slightly increases for 12 GPa to 0.60%/2.520 g/cm^3^. Densification at 15 GPa maximum pressure had a drastic influence on the glass and caused a densification of 2.08%/2.557 g/cm^3^. As previously mentioned, the 19 GPa densification was repeated with a smaller cylinder (19 GPa II) as the cylinder of the consecutive series (19 GPa I) was damaged while preparation. Sample 19 GPa I shows the greatest densification with 3.67% and a density of 2.597 g/cm^3^, within the series, while sample 19 GPa II shows lower densification at 3.15%/2.584 g/cm^3^. Higher densification of glass can occur due to the presence of shear stress as it can lead to a better folding of the glass network [30,31]. The increased densification value of 19 GPa I is ascribed to shear stress since the sample happened to be directly in contact with MgO without the NaCl pressure medium in between. The differences within the 19 GPa samples gives insight on the reproducibility of the densification process. It is found to be satisfying, as the 19 GPa I sample is within the error bars of the 21 GPa sample (3.55%/2.594 g/cm^3^) and at the fitted saturation of densification of 3.55 ± 0.14%. As revealed by the concomitant floatation measurement, the sample at 21 GPa is undoubtfully denser than the 19 GPa II. The fitting of the experimental values (black dashed-dotted curve) was done using an enhanced sigmoidal function (Equation (5)) which is based on a function (Equation (4)) proposed by Ji etal. [6]. The fitting parameters are the maximum densification *α*, a variable fitting parameter *β* and *P*_0_.

(4) $$\frac{∆\rho }{\rho_{0}}=\alpha \left[\frac{1}{1+\beta *e^{\left(\frac{-P}{P_{0}}\right)}}-\frac{1}{1+\beta }\right]$$

Equation (4) does not give direct access to physically meaningful variables. The correction term 1/(1 + *β*) rescales the value to zero at 0 GPa but then does not scale properly the full variation, so *α* is not the total variation if *β* is small. We propose to rewrite Equation (4) as followed for any variable *f* (Equation (5)).

(5) $$f\left(P\right)\;=\;\frac{∆}{1-\frac{1}{1+e^{4mP_{i}}}}\left[\frac{1}{1+e^{-4m(P-P_{i})}}-\frac{1}{1+e^{4mP_{i}}}\right]+f\left(P_{0}\right)$$

In that case Δ is the maximum variation, *P_i_* is the pressure at the inflection point and *m* is the slope at the inflection point for the normalized variation. For better comparison and general comprehension regarding the sensitivity of each method, it is also convenient to define the value *m^f^*, which is the slope at the inflection point using the unit of the variable *f*. It is uncomplicated to pass from Equation (4) to Equation (5) using the following equations:

(6) $$m=\frac{1}{4P_{0}}$$

(7) $$m^{f}=\frac{\alpha }{4P_{0}}=\frac{m\;∆}{1-\frac{1}{1+e^{4mP_{i}}}}$$

(8) $$P_{i}=P_{0}ln\beta$$

(9) $$∆=\alpha \left(1-\frac{1}{1+\beta }\right)$$

The sigmoidal formalism is a suitable mathematical representative to describe the evolution of densification of soda–lime glass. Anyway, with such an equation it is impossible to define a clear elastic threshold. An alternative, also used by Deschamps et al., is to approximate the data as a linear variation between two pressures *P*_1_ (≙*P_onset_*) and *P*_2_ (≙*P_end_*). *P*_1_ could then define the onset of the densification [9]. The dependence of the density or any variable *f* can then be written as the following interval Equation (10):

$$0\;<\;P\;<\;P_{\mathrm{onset}}\;\;f\left(P\right)\;=f\left(0\right)$$

(10) $${P_{\mathrm{onset}}}_{\;}<\;P\;<\;P_{\mathrm{end}}\;\;f\left(P\right)\;=m^{f}\;\left(P-P_{onset}\right)\;+\;f\left(0\right)$$

$$P_{\mathrm{end}}\;<\;P\;\;f\left(P\right)\;=\;f\left(0\right)\;+\;∆$$

where $P_{onset}=P_{i}-\frac{∆}{2m^{f}}\left(1-\frac{1}{e^{4mP_{i}}}\right)$ and $P_{end}=P_{i}+\frac{∆}{2m^{f}}\left(1+\frac{1}{e^{4mP_{i}}}\right)$.

When comparing the results of this study to the previously reported values for similar window glass compositions (blue Rouxel et al.; orange Kato et al.) and calibration curves (red dashed line, Ji et al.), which also were generated using MAP for densification, some significant differences can be seen. In the beginning of the densification process, only small variation occurs until reaching an onset. While the literature values are in good agreement to each other and report an onset of densification at *P_onset_* = 10.1 GPa, the onset determined in this study is located slightly higher at *P_onset_* = 10.9 ± 0.8 GPa, suggesting that the elastic response of this study is taking place over a slightly larger region. Beyond the onset, the most striking difference between the densification saturation level is obvious. The maximum densification a soda–lime–silicate glass was reported to be 6.3% being reached at *P_end_* = 19.0 GPa maximum pressure by Ji et al. and Rouxel et al. [6,10,11]. In this study a much lower densification saturation level with Δ = 3.55 ± 0.14% was determined at an earlier *P_end_* = 18.1 ± 0.8 GPa. In fact, the position of the inflection point *P_inflection_* = 14.5 GPa did not evolve between this study and the previous ones, but the slope *m* is higher (this work: *m* = 0.14 ± 0.02, literature: *m* = 0.11). The maximum densification observed by Kato et al. could be in good agreement with the maximum densification observed here. The fact that it was measured at a much lower pressure, suggests that the hydrostaticity of their experiment was significantly lower compared to this study [31]. The role of the difference of composition between the OptiWhite^TM^ used in this study and the Planilux^®^ used in the previous study cannot be completely ruled out, especially since the exact composition is not reported. However, since the glass properties do not vary drastically in the region of window glass, it is assumed that it wouldn’t majorly impact the calibration [32]. As the densification is a direct outcome of the atomic packing, the sigmoidal behavior is expected to be applicable to the spectroscopic observables as they are strongly affected by changes of the atomic structure, as well. Due to that, sigmoidal Equation (5) seems to be a reasonable choice and was applied to fit all data obtained in the spectroscopic experiments, except the Brillouin shift. All sigmoidal fitting parameters in terms of the centroid *σ* are given in Table 1.

### 4.2. Permanent Densified Soda–Lime Glass Characterization

#### 4.2.1. Raman Spectroscopy: Glass Structure Modification

**Figure 8:**
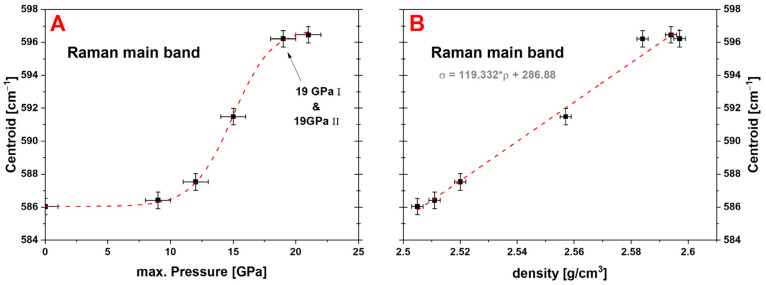
Evolution of the centroid of the Raman main band versus the maximum pressure (**A**) and the measured density (**B**); a variation of ~10 cm^−1^ can be observed from the pristine glass sample (~586 cm^−1^) to the maximal densified sample at 21 GPa (~596 cm^−1^); a fitted maximal variation Δ = 10.82 ± 0.15 cm^−1^ with *P_inflection_* = 14.9 ± 0.1 GPa and *m* = 0.16 ± 0.01 is reported in relation to *P_max_*, a linear dependency between the centroid and the density can be deduced (relation is given in the corresponding graph).

**Figure 9:**
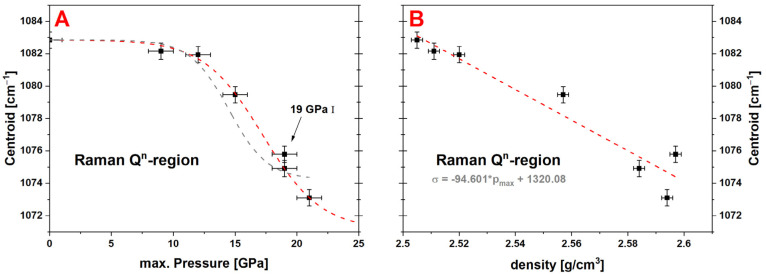
Evolution of the centroid of the Q^n^-region versus the maximum pressure (**A**) and the measured density (**B**); a variation of ~10 cm^−1^ can be observed from the pristine glass sample (~1083 cm^−1^) to the maximal densified sample at 21 GPa (~1073 cm^−1^); the sigmoidal fitting parameters are determined at Δ = −11.69 ± 1.03 cm^−1^, *P_inflection_* = 17.2 ± 0.6 GPa, *m* = 0.11 ± 0.01 (red dashed line), the gray dashed line is a force fit where *P_inflection_* was fixed at 14.5 GPa to display that the Q^n^-region shows varying behavior in comparison to the densification calibration; a linear dependency between the centroid and the density can be deduced (relation is given in the corresponding graph).

Figure 2 shows the overall measured Raman spectra of the experimental series from 400 cm^−1^ to 1300 cm^−1^, with the limits of the main band (500 cm^−1^–730 cm^−1^) marked by gray dashed lines. To better display the variations in the main band, Figure 3A highlights the isolated region. The main band consists of two contributions which vary over the experimental series: the contribution at ~560 cm^−1^ decreases while the contribution at ~600 cm^−1^ increases. Figure 8A shows the evolution of the centroid versus the maximum pressure and the density (Figure 8B). The centroid was determined at 586.0 cm^−1^ for the pristine glass and only a change within the error bars to 586.4 cm^−1^ can be observed for the 9 GPa sample. The 12 GPa sample shows the first significant variation to 587.5 cm^−1^, again suggesting that the limits of the elastic region falls between 9 GPa and 12 GPa. The 19 GPa and 21 GPa densified samples almost superimpose with a centroid value *σ* around 596.3 cm^−1^. These steady values state that the maximal closing of the Si–O–Si angle of the glass structure is reached. As the data seems to present a saturation at high pressure, it follows a similar sigmoidal behavior as previously observed for the densification. The centroid of the main band was fitted with Equation (5) (Figure 8A, red dashed line) and the obtained parameters are given in Table 1. The parameters *m* and *P_inflection_* are within the error bars of those determined for the densification. In Figure 9 the evolution of the Q^n^-region centroid is shown and a shift of ~10 cm^−1^ towards lower wavenumbers is reported starting at the centroid position of 1082.8 cm^−1^ at 0 GPa. The increase of *P_max_* to 9 GPa/1082.2 cm^−1^ and 12 GPa/1081.9 cm^−1^ showed no significant change on the centroid position, where at 15 GPa the centroid was determined to be 1079.5 cm^−1^. At higher pressures the centroid was evaluated at 1075.8 cm^−1^ for 19 GPa I, 1074.9 cm^−1^ for 19 GPa II and 1073.1 cm^−1^ at 21 GPa and does not show a saturation plateau. It shifts in the opposite direction compared to the main band. As there is still a significant variation at very high pressure, it can be deduced that even though no further densification is achieved in this study, the glass structure and its building groups are still responding to an increase of *P_max_* (red dashed line). The sigmoidal fit of this Q^n^ centroid gives *m* of 0.11 ± 0.01 and *P_inflection_* at 17.2 ± 0.6 GPa (Figure 9A). For comparison, the gray dashed line represents a force-fit with *P_inflection_* fixed at 14.5 GPa, which is the inflection point determined in the densification calibration. It is clear to see that the fit is not respecting the data at very high pressure and underestimates the saturation level. The calibration describing the dependency at the maximum pressure could be extended even further above 21 GPa when utilizing the Q^n^-region as observable. The Q^n^-region centroid shows an overall linear behavior in dependency to the measured density (Figure 9B), but with a higher dispersion compared to the main band behavior especially at higher densifications (Figure 8B).

**Figure 10:**
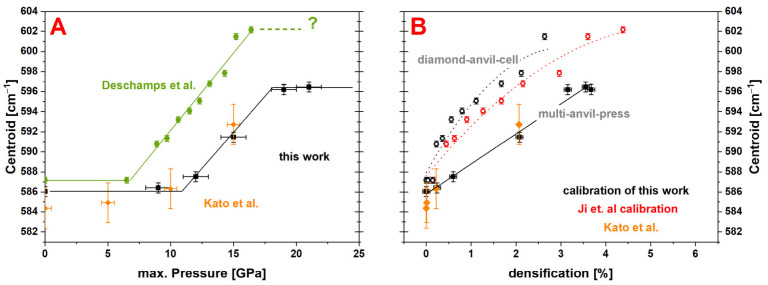
(**A**) Comparison of the Raman main band centroid evolution over the maximum pressure (black ≙ this work; green ≙ Deschamps et al.; orange ≙ Kato et al.); good overall agreement between the study of Kato et al. (MAP-densification) and the values determined in this work with the first structural variation starting at *P_onset_* = 11.8 ± 0.3 GPa and a plateau after *P_end_* = 18.0 ± 0.3 GPa at ~596 cm^−1^; values reported by Deschamps et al. show an earlier structural variation around 7 GPa and higher saturation value of ~602 cm^−1^; the discrepancy to Deschamps et al. could originate from the less hydrostatic conditions of the DAC used for compression. (**B**) Comparison of calibrations done with the Raman main band region (black ≙ this work; orange ≙ Kato et al.); filled symbols represent direct calibrations using MAP and are equivalent and linear; hollow symbols and guide for the eye (dotted curves) are generated by using the centroid data of Deschamps et al. (DAC) and the densification *P_max_* calibration of this work (black) respectively the calibration of Ji et al. (red).

Figure 10A shows the comparison of the Raman main band centroid to other studies on the dependency of the maximal pressure. The before-mentioned simplified linear approach was chosen for a better comparability with a determined *P_onset_* = 11.8 ± 0.3 GPa and *P_end_* = 18.0 ± 0.3 GPa. The values of this work are in good overall agreement with the values of Kato et al. (orange datapoints), even though the densification of the samples was determined to be much larger in comparison [7]. An obvious discrepancy to the study of Deschamps et al. (green datapoints) is evident [9]. An earlier structural variation with maximum pressure is visible around 7 GPa, which ends in a higher saturation value of ~602 cm^−1^. The differences can be explained by the densification conditions, as a DAC was used for compression in their experiments introducing more shear stress on the sample. This shear stress affects the glass network drastically as it effectively changes the Si–O–Si bond angles. The drastic effect of hydrostaticity was also observed for a-SiO_2_ [8]. The comparison of the Raman main band region of the calibration made by Ji et al. (red datapoints) and calibration of this work (black datapoints), as well as the results of the experiments made by Kato et al. (orange datapoints), in relation to the densification are shown in Figure 10B. Filled symbols represent experiments where the densification process was done using MAP. The newly proposed calibration curve is in close agreement to the values reported by Kato et al. The effect of using our new calibration instead of the previous calibration is illustrated also in Figure 10B. As direct access to the density was not possible in their experiments, Deschamps et al. deduced the densification from their observation of the main band centroid by applying the calibration given by Ji et al. (reported here in Figure 7). Their observations are reported in Figure 10B using both calibrations. The above-mentioned difference of several percent in densification is logically visible. None of them lead to a good agreement with this study (black line). It is clear to see that the spectral changes of the main band follow a non-linear behavior in DAC experiments. This is a direct consequence of the non-sigmoidal shape of their observations.

#### 4.2.2. Brillouin Spectroscopy

**Figure 11:**
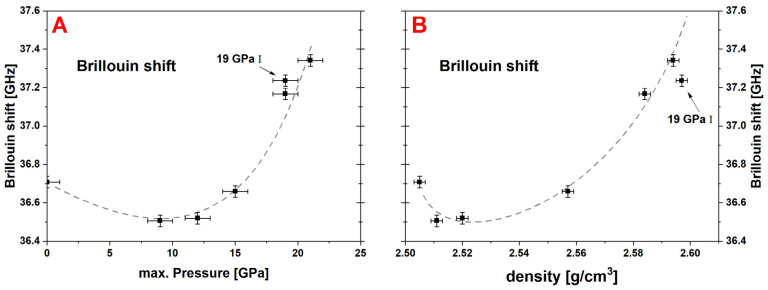
Evolution of the Brillouin shift of the permanently densified glass samples versus the maximum pressure (**A**) and the measured density (**B**); dashed gray lines are guide to the eye; a non-linear evolution is evident with a decrease of the Brillouin shift at moderate densification and a steady increase to the highest applied *P_max_*; a similar behavior with a minimum of the elastic properties was observed on permanently densified amorphous silica, but not reported for soda–lime–silicate glass up to now.

The Brillouin shift along the sample series presents an obvious non-linear evolution (Figure 11). It decreases unexpectedly from 36.71 GHz to 36.51 GHz with the first applied *P_max_* at 9 GPa and a densification of ~0.4%. After this minimum, it increases with maximal pressures up to 37.34 GHz at 21 GPa without showing any plateau. This last fact suggests that the long-range elastic properties are still affected by exceedingly applied pressure. Densified silica glass at ~4% densification also presents a minimum of its longitudinal sound velocity [8,33]. Local polyamorphism and the coexistence of low-density-amorphous and high-density-amorphous states were proposed to explain this phenomenon [34,35]. Anyhow, to our knowledge this was not reported for soda–lime–silicate glass, but the obtained experimental data implies that the structure of permanently densified soda–lime–silicate glass is presenting similar phenomena.

#### 4.2.3. Nd^3+^ and Eu^3+^ Luminescence

**Figure 12:**
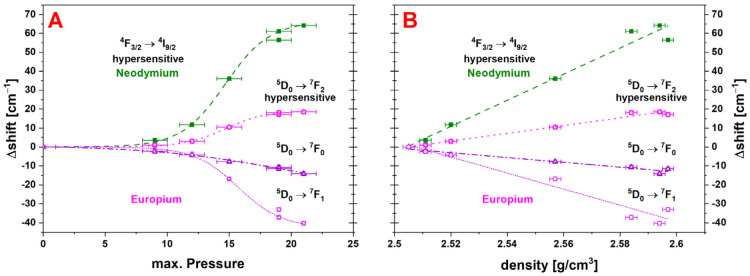
(**A**) Comparison of the different evaluated transitions of the luminescence of Nd^3+^ (^4^F_3/2_ → ^4^I_9/2_ ≙ green squares/dashed) and Eu^3+^ (pink hexagons/short dashed ≙ ^5^D_0_ → ^7^F_2_; lavender circles/dotted ≙ ^5^D_0_ → ^7^F_1_, purple triangles/dashed-dotted ≙ ^5^D_0_ → ^7^F_0_) versus applied *P_max_*. (**B**) Comparison of the different evaluated transitions of the luminescence of Nd^3+^ and Eu^3+^ with changing density; all transitions show a linear behavior and a rather large shift; the largest magnitude of shift is obtained for the hypersensitive transition of the Nd^3+^ with a fitted variation Δ = −66.31 ± 0.65%, REE-luminescence is considered as a high-potential intrinsic density sensor in glass.

The shift of the centroid of the hypersensitive ^4^F_3/2_ → ^4^I_9/2_ transition of Nd^3+^-luminescence as well as the centroid of the three electronic transitions of Eu^3+^ are reported versus *P_max_* and density respectively in Figure 12A,B. The nominal measured positions of these centroids are given in the appendix Table A3. All analyzed transitions have a sigmoidal dependence with *P_max_* and the results of the fit are listed in Table 1. The parameters obtained for the hypersensitive transition ^4^F_3/2_ → ^4^I_9/2_ of Nd^3+^ and ^5^D_0_ → ^7^F_2_ of Eu^3+^ are very similar to each other, as well as when compared to the fitting parameters of the densification evolution (Table 1). In comparison, the ^5^D_0_ → ^7^F_1_ transition of Eu^3+^ has a *P_inflection_* 1 GPa higher and shows a less pronounced saturation plateau. This trend is even more distinct for the ^5^D_0_ → ^7^F_0_ transition of Eu^3+^, for which the sigmoidal fit does not apply properly and therefore leads to very large error bars on the fitting parameters. The dependence of all the luminescence centroids with density is satisfactory linear (Table 2). For the samples densified with greater *P_max_* the dispersion is larger, which means that a further increase of the maximum pressure might influence the atomic environment of the Nd atoms in a more complicated way.

### 4.3. Comparison and Sensitivity Assessment

**Figure 13:**
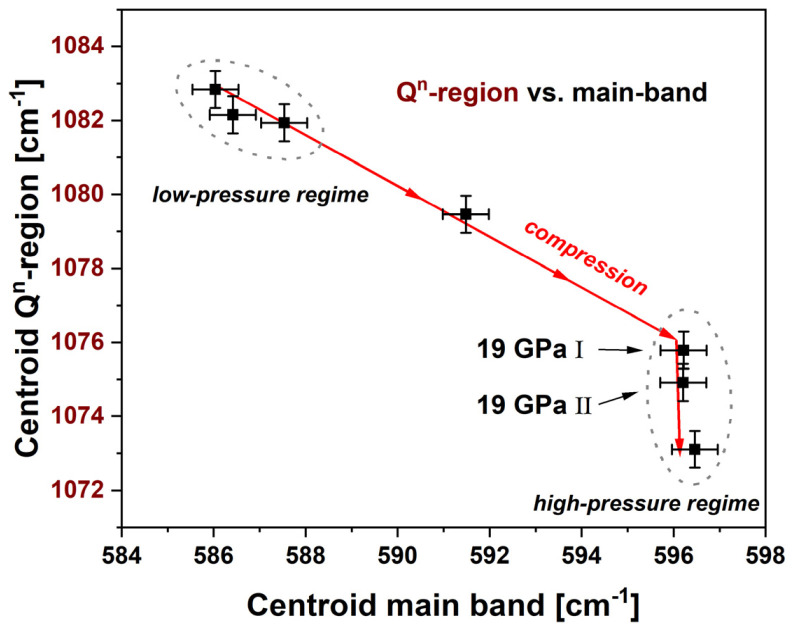
Comparison of the centroid of the Q^n^-region (left red axis) vs. the main band centroid (bottom black axis); a stronger variation of the main band in the elastic region can be observed; a linear evolution in the plastic deformation region up to 19 GPa is evident as a logical follow up as both observables behave in the same way in this region; above 19 GPa the Q^n^-region still shows variation while the main band remains the same; when utilizing Raman spectroscopy to analyze the pressure history it is preferable to use the main band calibration in the elastic region from moderate pressure up to its *P_onset_* = 11.8 ± 0.3 GPa and the Q^n^-calibration at very high pressure as its *P_end_* = 21.7 ± 1.0 GPa is located higher; in the plastic deformation region between *P_onset_* < *P_max_* < *P_end_* both calibrations are identical, but the main band shows a higher *m^f^* = 1.73 ± 0.13 cm^−1^ (Q^n^-region: *m^f^* = −1.29 ± 0.01 cm^−1^) and therefore is advantageous.

The spectroscopic methods were compared to each other to assess their sensitivity at the various pressure regions and give an estimation of their applicability. Since the instrumental precision is similar for the Raman and luminescence centroids, the sensitivities of the proposed calibration equations are then directly related to the slope at the inflection point *m^f^*, which takes into account both the magnitude of the maximal variation Δ, and its steepness *m*. It is then clear that the luminescence data permits the more precise calibrations. The best is the hypersensitive emission of Nd^3+^ corresponding to the transition ^4^F_3/2_ → ^4^I_9/2_ with a *m^f^* of −9.3 cm^−1^, followed by the ^5^D_0_ → ^7^F_1_ Eu^3+^ transition with 6 cm^−1^. Between the two studied Raman contributions, the main band has a better sensitivity compared to the Q^n^-region, since *m^f^* is respectively of 1.7 cm^−1^ and −1.3 cm^−1^. Most of the spectroscopic data are directly proportional to the density evolution, i.e., resulting in similar sigmoidal fitting parameters, though still some slight differences exist which then could infer more information. The more obvious divergence from the densification behavior is found in the evolution of the centroid of the Q^n^-region with a *P_onset_* of 1.8 GPa and a *P_end_* of 3.6 GPa above that of the densification. Both observables of the Raman spectra are plotted against each other in Figure 13, where the evolution of the main band is synchronous with densification. The presence of a high- and a low-pressure structural regime can be identified. For samples densified with *P_max_* < 19 GPa, a linear correlation is obtained between the two observables. This seems to indicate that the Si–O–Si angles are changed in the same way as the sum of the variation of the Q^n^-species and the distance of Si–O during densification. Above 19 GPa the Q^n^-region still shows variation, i.e., translation from Q^3^- to Q^2^-species, while the main band does not evolve anymore. This suggests that the angle of the Si–O–Si bonding is locked in the closest possible position, but a reorganization of the building groups is still effective, whereas no further large-scale densification is achieved. Subsequently, only the Q^n^-region calibration is able to resolve changes at very high maximum pressures when utilizing Raman spectroscopy.

**Figure 14:**
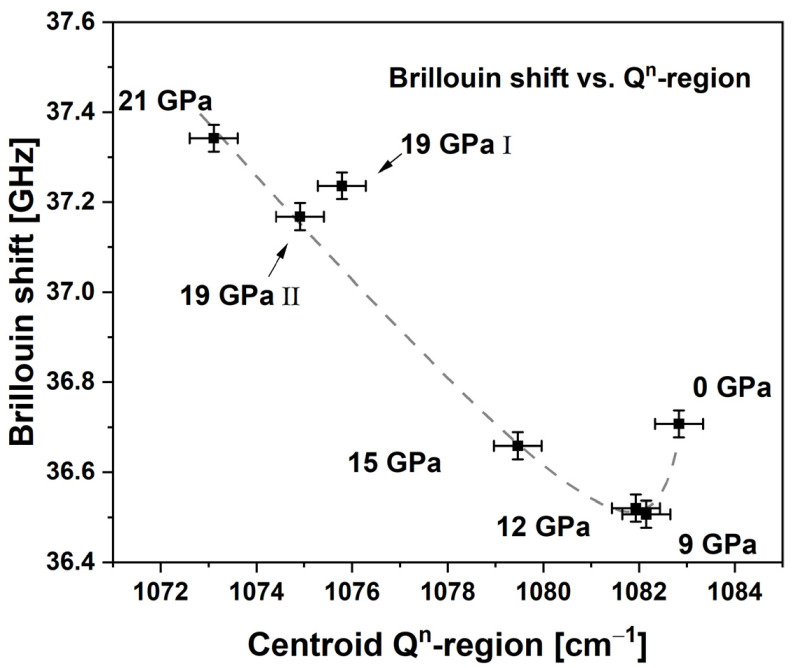
Comparison of the Brillouin shift with the centroid of the Raman Q^n^-region (gray dashed line is only a guide to the eye); using this cross spectral observation the samples densified >9 GPa are almost aligned and the non-hydrostatical sample at 19 GPa I in seen to be clearly out of trend.

Utilizing the Brillouin shift to determine the density or the maximum pressure encountered is more complex and needs to be assessed carefully. As seen in Figure 11, due to the non-linear behavior of the Brillouin shift at moderate *P_max_*, a measured Brillouin shift of 36.7 GHz can correspond to pristine glass or to a glass densified by ~2.4% at *P_max_* ~ 16 GPa. The non-linear behavior of the Brillouin signal is very surprising, since it is not seen on any other studied observable. The best correlation is obtained if the Brillouin shift is compared to Q^n^ centroid (see Figure 14). All the samples are almost linear except for the pristine glass and the non-hydrostatical sample 19 GPa I. Using such a cross-spectroscopic approach could be useful in distinguishing the stress deviatoric during densification. The underlying atomic explanation of this correlation needs to be elucidated.

The argued sensitivity of the densification and structural modification due to the non-hydrostatic conditions were used to explain the earlier densification of the 19 GPa I sample, as well as the discrepancy of this study with the DAC study from Deschamps et al. and needs to be carefully addressed in the future to improve the reliability of the applicability of these calibrations to interpret mappings of samples. Crossing the information obtained by REE-luminescence emission with Raman and/or Brillouin spectroscopy is a very promising way to go deeper with data interpretation.

## 5. Conclusions

In this article, calibration curves for several spectroscopic approaches were presented with the purpose of assessing the local pressure history and densification of glass at the micrometer scale. With the given detailed information of the procedure of spectroscopic data treatment and the provided calibrations, the direct application of these calibrations is made possible to the reader. A maximal densification for soda–lime–silicate glass of Δ = 3.55 ± 0.14% was determined, which is significantly lower than the values reported in the literature. This discrepancy could be explained by the high precision needed for density measurements or by different hydrostatic compression conditions during sample preparation. As shown with the sample that was in direct contact with MgO, any divergence from hydrostaticity induces an onset of densification at lower pressures. The evaluated centroid parameters showed an overall linear behavior in relation to the high precision determined density of the sample series for all spectroscopic approaches. Brillouin spectroscopy revealed a non-linear evolution at moderate pressures, suggesting a structural reorganization far before the densification onset pressure, showing similarities with vitreous SiO_2_. A detailed discussion and interpretation about the structural origin of the spectroscopic variations will be made in a separate article. It was shown that the REE-luminescence of Nd^3+^ and Eu^3+^ evolved with a larger amplitude and a rather short acquisition time in comparison to Raman and Brillouin spectroscopy. Their high sensitivity allows the detection of even rather small changes of local pressure or density. Due to the natural abundance of Nd_2_O_3_ in soda–lime glass or uncomplicated doping of the glass melt with ppm of Eu_2_O_3_ and the above-mentioned benefits, REE-luminescence can function as an ideal intrinsic sensor and is highly recommended for spectroscopic assessment of the local pressure history and local densification.

## Figures and Tables

**Table 1:** Sigmoidal fitting parameters (Equation (5)) for densification and various spectroscopic methods in relation to the centroid *σ* versus *P_max_*.

Densification	Δ	*m*	*m^f^*	*P_inflection_*	*P_onset_*	*P_end_*
	%	[a.u.]	%	[GPa]		
**This work**	3.55 (±0.14)	0.14 (±0.02)	0.50 (±0.09)	14.5 (±0.3)	10.9 (±0.8)	18.1 (±0.8)
**Ji et al.** [6] **^†^**	6.3	0.11	0.71	14.5	10.1	19.0
**Spectroscopic Method**	**[cm^−1^]**	**[a.u.]**	**[cm^−1^]**	**[GPa]**		
**Raman main band**	10.82 (±0.15)	0.16 (±0.01)	1.73 (±0.13)	14.9 (±0.1)	11.8 (±0.3)	18.0 (±0.3)
**Raman Q^n^-region**	−11.69 (±1.03)	0.11 (±0.01)	−1.29 (±0.01)	17.2 (±0.6)	12.7 (±1.0)	21.7 (±1.0)
**Centroid Nd^3+^ → ** **^4^** **F_3/2_ → ^4^** **I_9/2_**	−66.31 (±0.65)	0.14 (±0.01)	−9.29 (±0.57)	14.7 (±0.1)	11.1 (±0.4)	18.3 (±0.4)
**Centroid Eu^3+^ → ^5^D_0_ → ** **^7^F_2_**	−19.10 (±0.35)	0.15 (±0.01)	−2.87 (±0.14)	14.7 (±0.1)	11.4 (±0.3)	18.0 (±0.3)
**Centroid Eu^3+^ → ^5^D_0_ → ** **^7^F_1_**	42.51 (±1.62)	0.14 (±0.02)	5.95 (±1.08)	15.7 (±0.3)	12.1 (±0.8)	19.3 (±0.8)
**Centroid Eu^3+^ → ^5^D_0_ → ** **^7^F_0_**	24.12 (±15.30)	0.05 (±0.02)	1.18 (±1.25)	19.3 (±6.4)	9.3 (±11.1)	29.7 (±11.1)

^†^ No error bars for the fitting parameters were given in reference.

**Table 2:** Linear fitting equations for the various spectroscopic methods to calculate density as function of the centroid *σ*; the error was estimated as the mathematical mean of the difference (*ρ_measured_* − *_calibration_*) of the cylinders densified at various *P_max_.*

Spectroscopic Method	Density	Error	R^2^
**main band**	*ρ* = 0.00828*σ − 2.35	0.004	0.988
**Q^n^-region**	*ρ* = −0.00980*σ + 13.12	0.009	0.927
**Centroid Nd^3+^ →** **^4^** **F_3/2_ → ^4^** **I_9/2_**	*ρ* = −0.00142*σ + 18.48	0.009	0.979
**Centroid Eu^3+^ → ^5^D_0_ → ** **^7^F_2_**	*ρ* = −0.00479*σ + 80.54	0.059	0.983
**Centroid Eu^3+^ → ^5^D_0_ → ** **^7^F_1_**	*ρ* = 0.00226*σ − 35.69	0.047	0.957
**Centroid Eu^3+^ → ^5^D_0_ → ** **^7^F_0_**	*ρ* = 0.00749*σ − 126.94	0.070	0.957

## Data Availability

The full data sets are contained within the article and the Appendix A.

## Appendix A

**Table A1:** Sigmoidal fitting parameters (Equation (5)) for densification and various spectroscopic methods in relation to the *mean* $\bar{I}$ and *P_max_*.

Densification	Δ	*m*	*m^f^*	*P_inflection_*	*P_onset_*	*P_end_*
	%	[a.u.]	%	[GPa]		
**This work**	3.55 (±0.14)	0.14 (±0.02)	0.50 (±0.09)	14.5 (±0.3)	10.9 (±0.8)	18.1 (±0.8)
**Ji et al.** [6] **^†^**	6.3	0.11	0.71	14.5	10.1	19.0
**Spectroscopic Method**	**[cm^−1^]**	**[a.u.]**	**[cm^−1^]**	**[GPa]**		
**Raman main band**	5.16 (±0.21)	0.16 (±0.03)	0.83 (±0.19)	14.6 (±0.3)	11.5 (±0.9)	17.7 (±0.9)
**Raman Q^n^-region**	−15.82 (±4.14)	0.08 (±0.02)	−1.27 (±0.01)	18.1 (±2.0)	11.9 (±3.6)	24.4 (±3.6)
**Mean Nd^3+^ → ** **^4^** **F_3/2_ → ^4^** **I_9/2_**	−50.93 (±0.93)	0.15 (±0.01)	−7.64 (±0.37)	14.7 (±0.1)	11.4 (±0.3)	18.0 (±0.3)
**Mean Eu^3+^ → ^5^D_0_ → ** **^7^F_2_**	−13.46 (±0.24)	0.15 (±0.01)	−2.02 (±0.10)	14.5 (±0.1)	11.2 (±0.3)	17.8 (±0.3)
**Mean Eu^3+^ → ^5^D_0_ → ** **^7^F_1_**	21.49 (±1.34)	0.12 (±0.01)	2.58 (±0.38)	16.1 (±0.4)	11.9 (±0.7)	20.3 (±0.7)
**Mean Eu^3+^ → ^5^D_0_ → ** **^7^F_0_**	21.94 (±3.25)	0.08 (±0.02)	1.71 (±0.68)	15.2 (±1.3)	8.8 (±2.9)	21.7 (±2.9)

^†^ no error bars for the fitting parameters were given in reference.

**Table A2:** Linear fitting equations for the various spectroscopic methods to calculate density as function of the ***mean*** $\bar{I}$; the error was estimated as the mathematical mean of the difference (***ρ_measured_** − **ρ_calibration_***) of the cylinders densified at various *P_max_.*

Spectroscopic Method	Density	Error	R^2^
**main band**	*ρ* = 0.01635*$\bar{I}$ − 7.18	0.004	0.987
**Q^n^-region**	*ρ* = −0.00892*$\bar{I}$ + 12.09	0.008	0.938
**Centroid Nd^3+^ → ^4^F_3/2_ → ** **^4^I_9/2_**	*ρ* = −0.00183*$\bar{I}$ + 23.06	0.018	0.970
**Centroid Eu^3+^ → ^5^D_0_ → ** **^7^F_2_**	*ρ* = −0.00686*$\bar{I}$ + 113.97	0.034	0.983
**Centroid Eu^3+^ → ^5^D_0_ → ** **^7^F_1_**	*ρ* = 0.00485*$\bar{I}$ − 79.49	0.060	0.953
**Centroid Eu^3+^ → ^5^D_0_ → ** **^7^F_0_**	*ρ* = 0.00529*$\bar{I}$ − 89.01	0.008	0.964

**Table A3:** Experimental and spectroscopic data (***centroid*** ***σ***) of the permanently densified glass cylinders.

P_max_	Density	Densification	Densification Sigmoidal-Fit	Centroid Main Band	Centroid Q^n^-Region	Brillouin Shift	Centroid Nd^3+^ ^4^F_3/2_ → ^4^I_9/2_ (Hypersensitive)	Centroid Eu^3+^ ^5^D_0_ → ^7^F_2_ (Hypersensitive)	Centroid Eu^3+^ ^5^D_0_ → ^7^F_1_	Centroid Eu^3+^ ^5^D_0_ → ^7^F_0_
[GPa]	[g/cm^3^]	[%]		[cm^−1^]	[cm^−1^]	[GHz]	[cm^−1^]			
±1	±0.002	±0.08	---	±0.5	±0.5	±0.03	±1.0			
0	2.505	0	0	586.0	1082.8	36.71	11,253.6	16,279.5	16,922.1	17,291.5
9	2.511	0.24	0.15	586.4	1082.2	36.51	11,250.1	16,278.5	16,924.6	17,293.7
12	2.520	0.60	0.70	587.5	1081.9	36.52	11,241.8	16,276.5	16,926.4	17,295.6
15	2.557	2.08	2.02	591.5	1079.5	36.66	11,217.4	16,269.1	16,939.0	17,299.3
19 I	2.597	3.67	3.29	596.2	1075.8	37.24	11,197.0	16,262.4	16,955.1	17,303.1
19 II	2.584	3.15	3.29	596.2	1074.9	37.17	11,192.4	16,261.4	16,959.2	17,302.2
21	2.594	3.55	3.46	596.5	1073.1	37.34	11,189.3	16,261.0	16,962.3	17,305.7

**Table A4:** Experimental and spectroscopic data (mean·$\bar{I}$) of the permanently densified glass cylinders.

P_max_	Density	Densification	Densification Sigmoidal-Fit	Mean Main Band	Mean Q^n^-Region	Brillouin Shift	Mean Nd^3+^ ^4^F_3/2_ → ^4^I_9/2_ (Hypersensitive)	Mean Eu^3+^ ^5^D_0_ → ^7^F_2_ (Hypersensitive)	Mean Eu^3+^ ^5^D_0_ → ^7^F_1_	Mean Eu^3+^ ^5^D_0_ → ^7^F_0_
[GPa]	[g/cm^3^]	[%]		[cm^−1^]	[cm^−1^]	[GHz]	[cm^−1^]			
±1	±0.002	±0.08	---	±0.5	±0.5	±0.03	±1.0			
0	2.505	0	0	592.2	1074.3	36.71	11,222.2	16,254.2	16,918.4	17,296.7
9	2.511	0.24	0.15	592.1	1072.7	36.51	11,220.0	16,253.4	16,920.0	17,299.1
12	2.520	0.60	0.70	593.1	1072.6	36.52	11,213.3	16,251.7	16,920.8	17,302.2
15	2.557	2.08	2.02	595.0	1069.9	36.66	11,194.8	16,246.6	16,926.4	17,308.0
19 I	2.597	3.67	3.29	597.7	1065.8	37.24	11,179.8	16,242.1	16,933.8	17,312.7
19 II	2.584	3.15	3.29	597.2	1065.0	37.17	11,174.0	16,241.4	16,935.6	17,312.3
21	2.594	3.55	3.46	597.1	1063.2	37.34	11,173.3	16,241.2	16,937.8	17,316.1
